# A Tumor Treated With Antibiotics: A Rare Case

**DOI:** 10.7759/cureus.32819

**Published:** 2022-12-22

**Authors:** Anas Mahmoud, Dawid Nowak, Arslan Chaudhry, Michael Agnelli

**Affiliations:** 1 Internal Medicine, St. Joseph’s University Medical Center, Paterson, USA; 2 Internal Medicine, St. Joseph's University Medical Center, Paterson, USA

**Keywords:** osteomyelitis, gram-positive bacteria, incision and drainage of abscess, omfs, anaerobe, frontal sinus, antibiotic usage, recurrent sinusitis, pott's puffy tumor

## Abstract

Pott's puffy tumor, a rare condition, is an osteomyelitis involving the frontal bone with accompanying subperiosteal abscess. Patients typically present with swelling of the scalp and forehead, headache, fever, tenderness of the frontal sinus, and nasal discharge. MRI is the modality of choice for diagnosis and assessment. The standard of care is incision and drainage with long-course antibiotics. The prognosis is excellent; however, complications from a hematogenous spread can lead to meningitis or epidural spaces if not treated properly.

## Introduction

Pott's puffy tumor (PPT) is a rare condition that occurs secondary to osteomyelitis of the frontal bone with subperiosteal abscess collection. PPT is an infrequent presentation in the older population, which can cause high morbidity if not diagnosed, managed, and followed up appropriately. PPT has a variable presentation ranging from a small to a large abscess and localized to spread behavior. Current guidelines outline management through antimicrobial and surgical intervention. Expert opinion from an otolaryngologist and oral and maxillofacial surgeon is recommended to avoid unnecessary complications [1]. The prognosis is excellent. PPT incidence has regressed thanks to antibiotics, with the highest incidence in adolescents infected with recurrent sinusitis. To our knowledge, we report the oldest patient with PPT who was successfully treated.

## Case presentation

**Figure 1:**
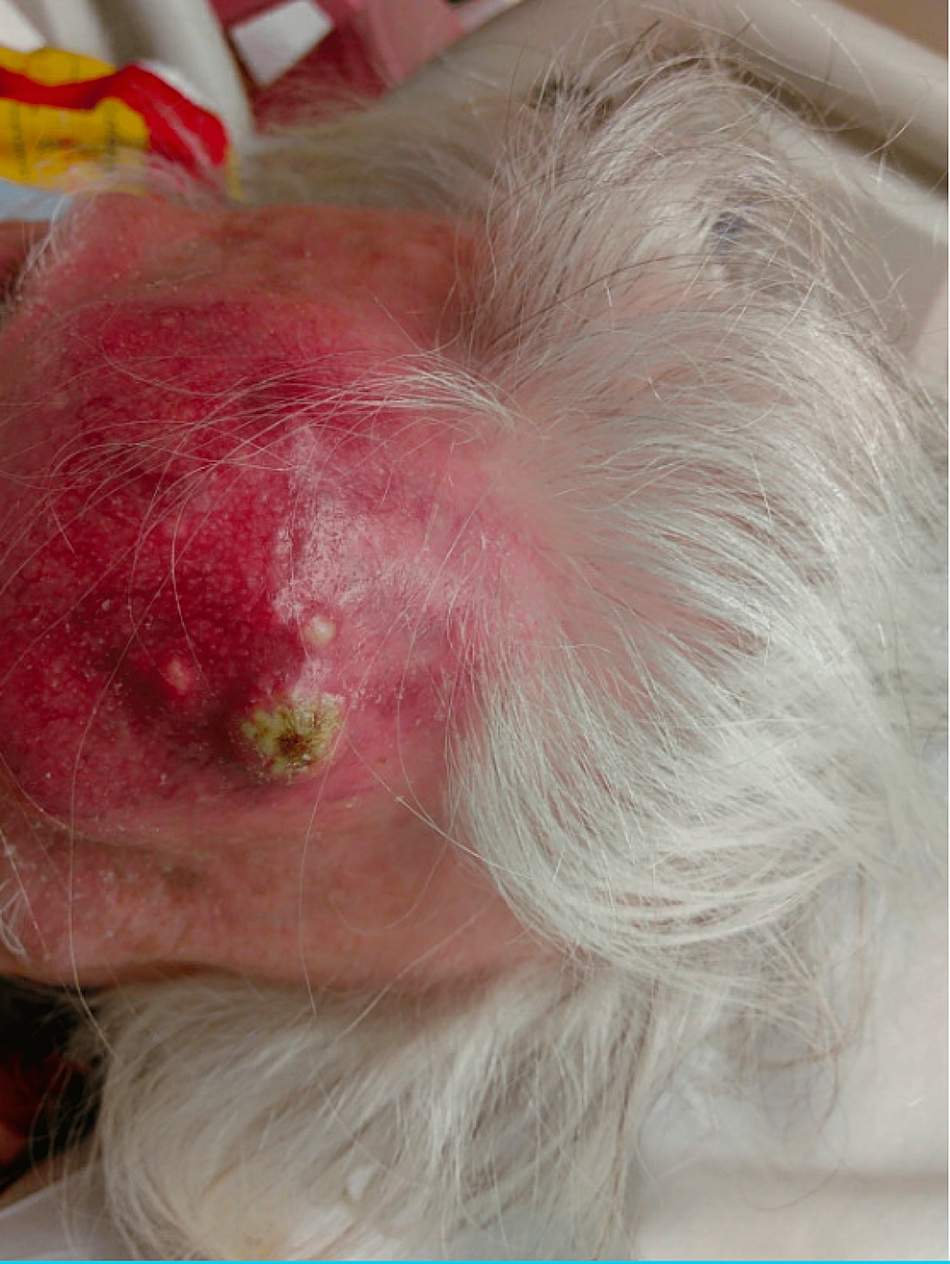
Pott's puffy tumor of the size of a softball protruding from the forehead.

**Figure 2:**
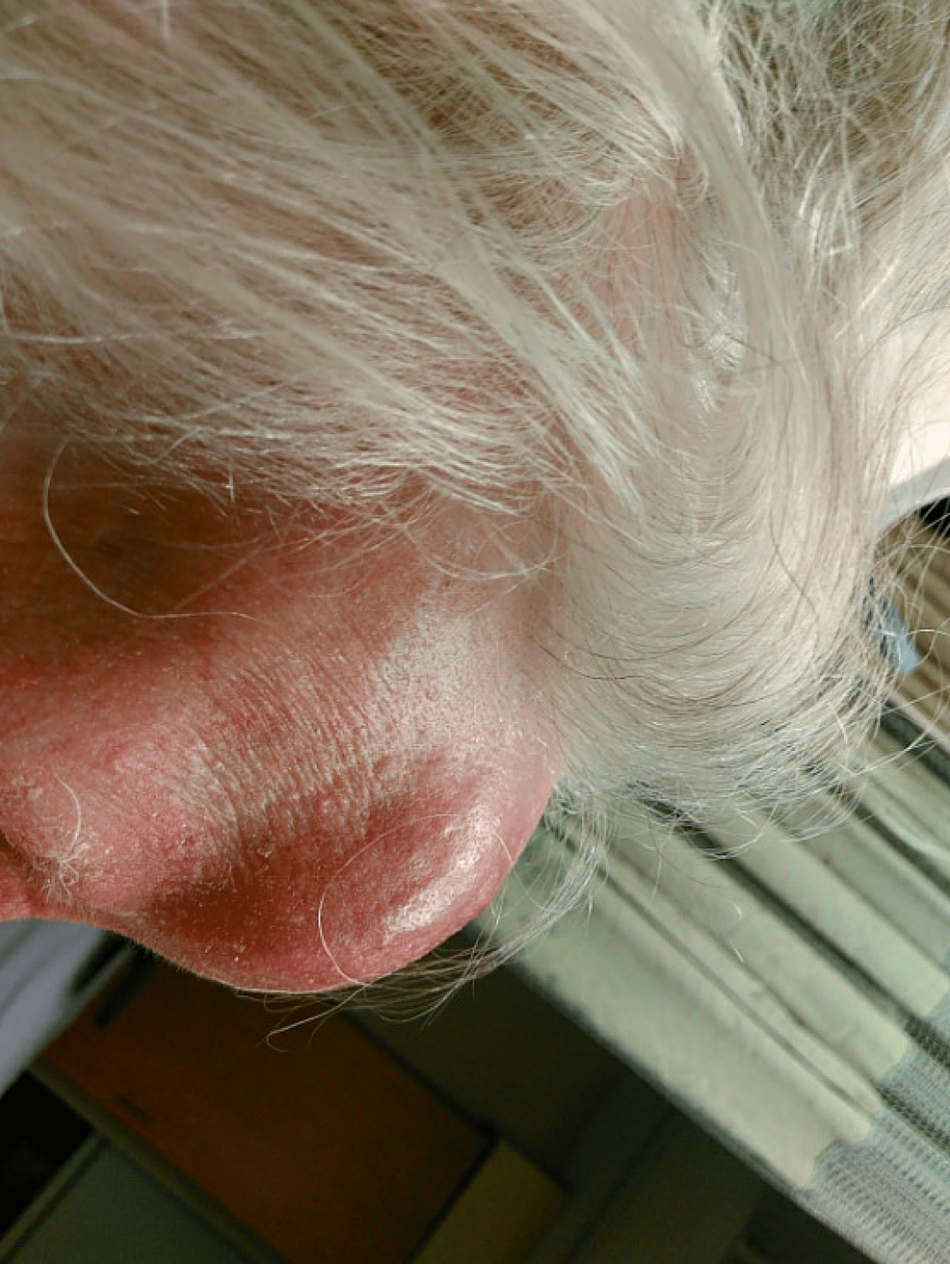
Side view of Pott's puffy tumor demonstrating the protrusion of the forehead mass.

**Figure 3:**
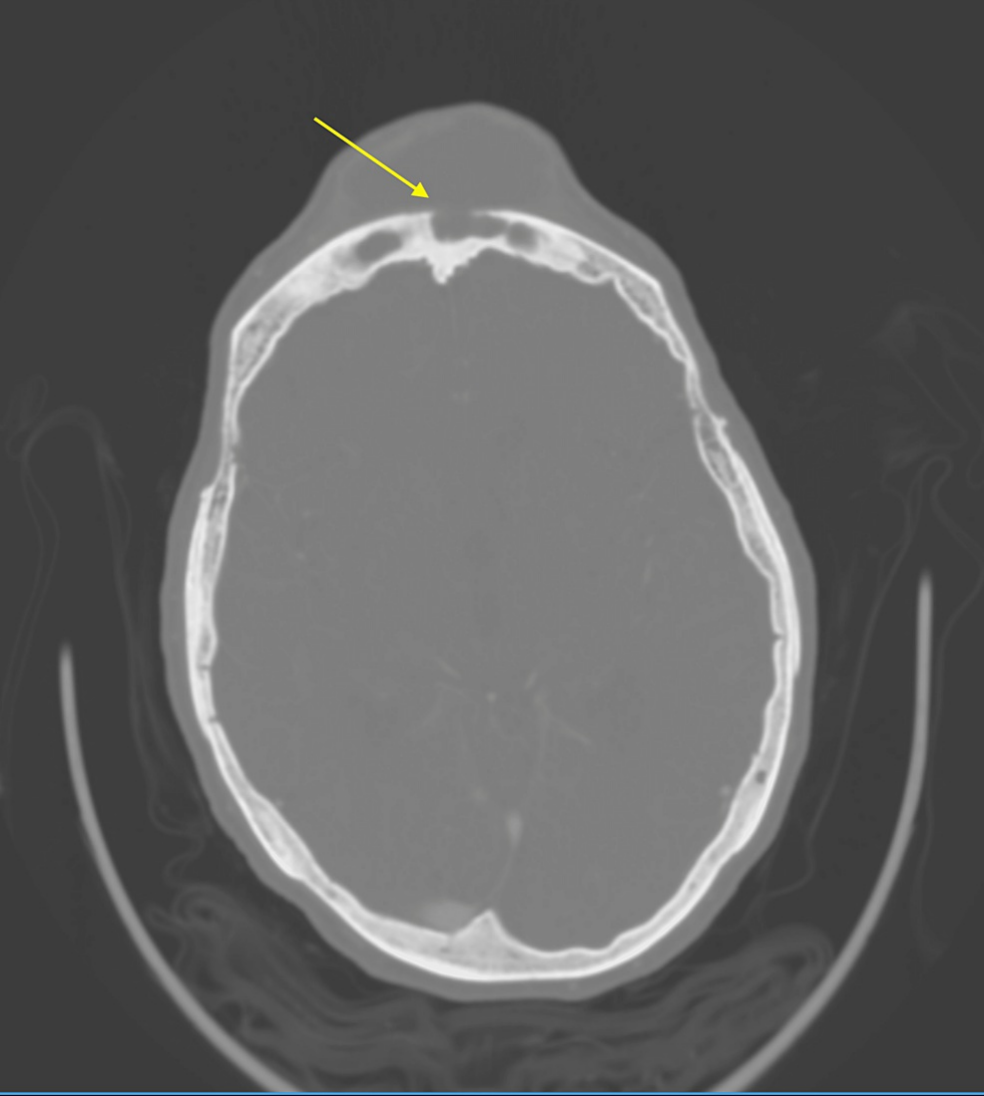
CT scan of the head showing a large, likely chronic subcutaneous collection in the midline frontal region with the erosion of adjacent frontal bone (yellow arrows) with possible communication to the left frontal sinus.

**Figure 4:**
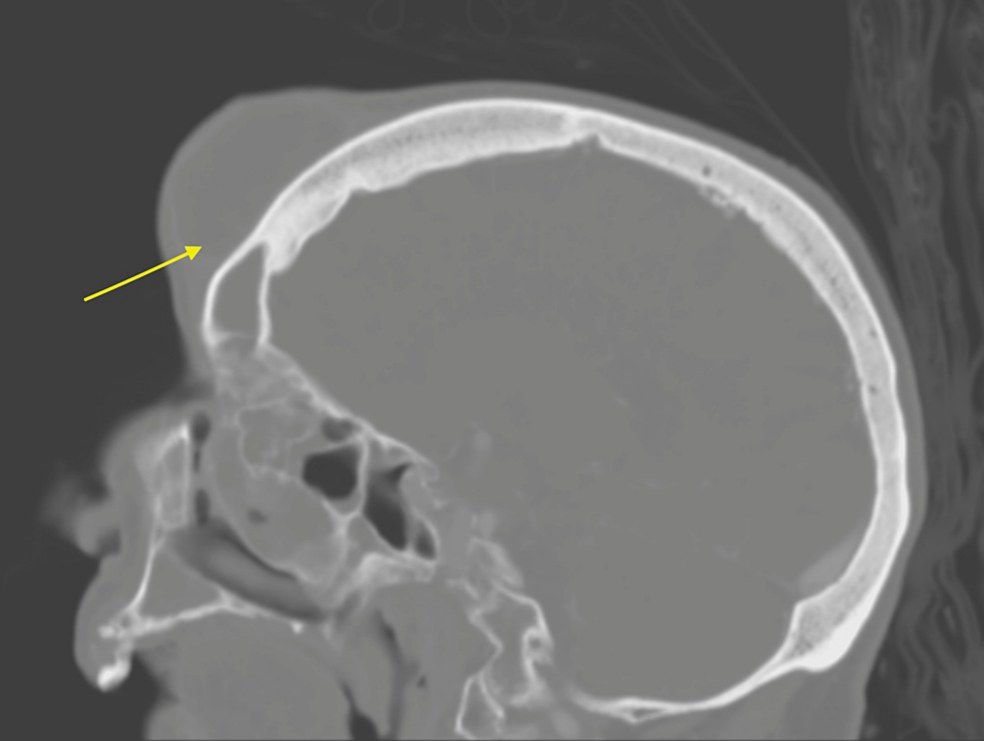
CT scan of the head showing a large, likely chronic subcutaneous collection in the midline frontal region (yellow arrow) with the erosion of adjacent frontal bone with possible communication to the left frontal sinus.

**Figure 5:**
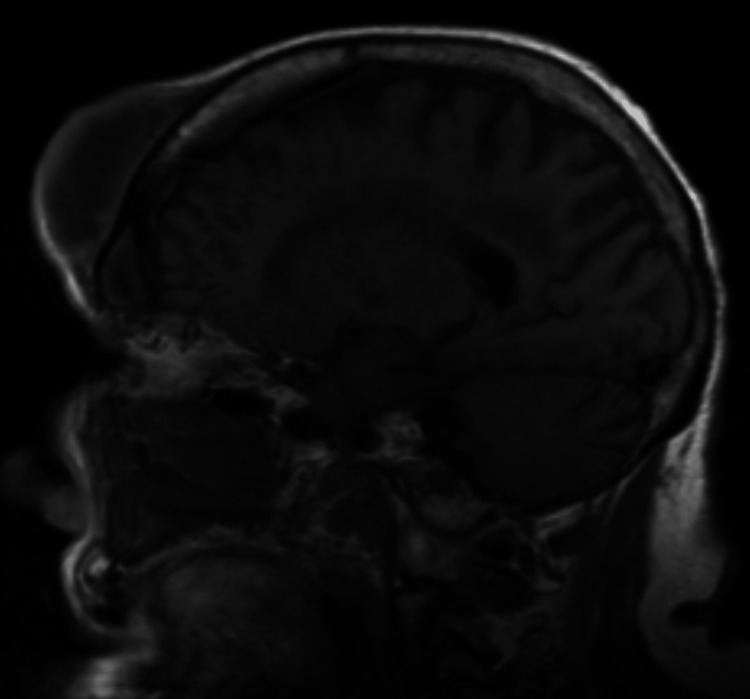
MRI of the brain showing bifrontal, maxillary, and ethmoid sinusitis. Bony erosion of the frontal calvarium extending into the bifrontal extra-axial collection. The findings are suggestive of abscess and frontal osteomyelitis with sinusitis.

**Figure 6:**
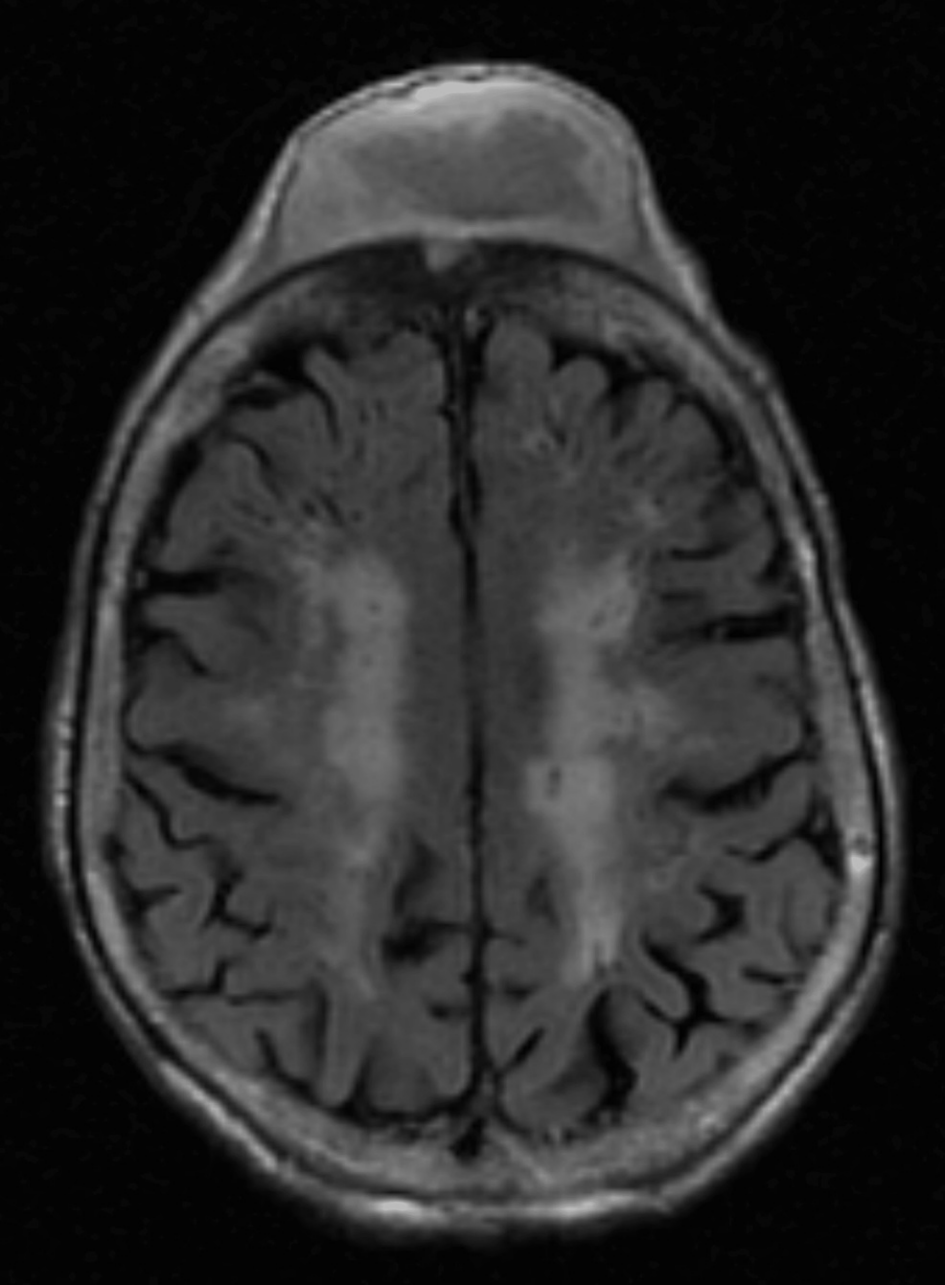
Bifrontal, maxillary, and ethmoid sinusitis. Bony erosion of the frontal calvarium extending into the bifrontal extra-axial collection. The findings are suggestive of abscess and frontal osteomyelitis with sinusitis.

**Figure 7:**
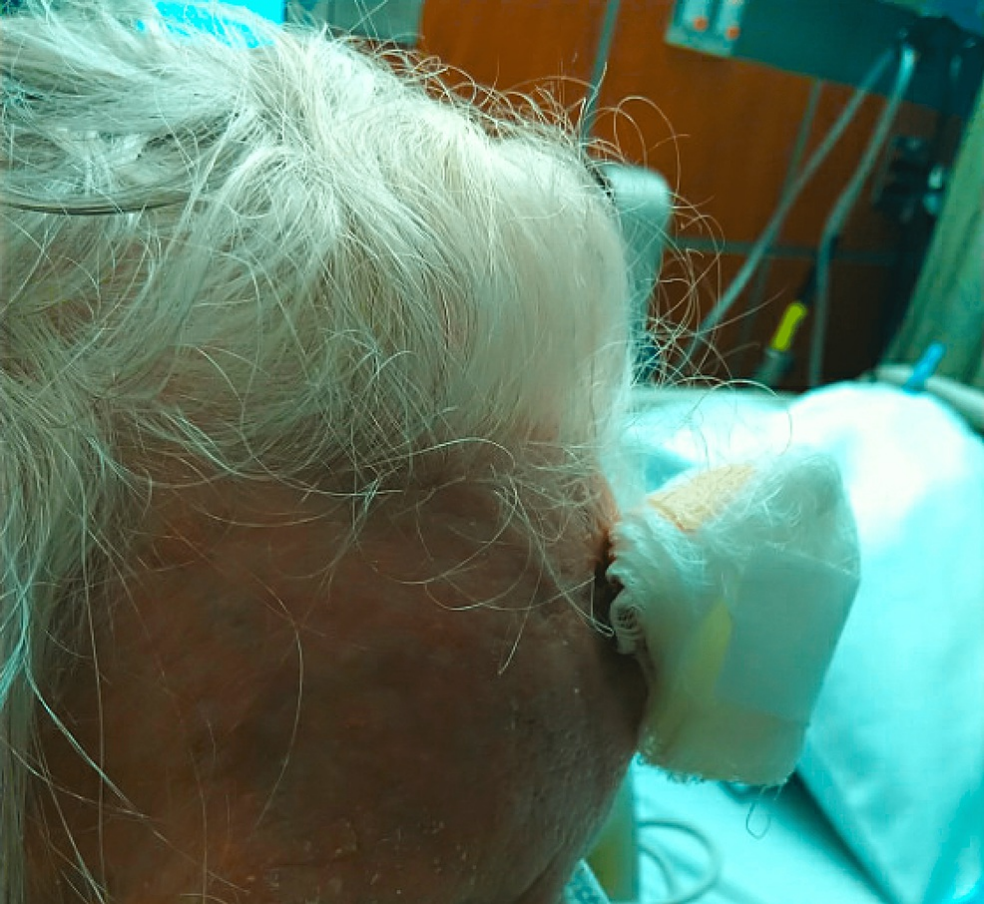
Resolution of the swelling one week after incision and drainage of the abscess.

The patient was a 92-year-old Polish female with a past medical history of tuberculosis (TB) who presented with a chief complaint of a forehead mass, which started one week prior to admission as a painful small golf ball-sized mass on her forehead and rapidly increased to a size of a softball over two nights. She denied any trauma or hitting her head recently. She did describe a similar episode of forehead swelling 30 years ago and she allegedly had the mass lanced. She denied any acting purulent drainage from the forehead. Of note, the patient was diagnosed with TB 50 years ago in Poland before she moved to the US. She does not remember the details of the treatment. Vitals were stable, the patient was afebrile, and the labs came back without significant abnormalities. Physical exam (Figures 1, 2) was remarkable for a large (5 x 6 cm), erythematous, tender, and fluctuant mass on her forehead. CT and MRI of the head (Figures 3-6) exhibited bifrontal, maxillary, and ethmoid sinusitis with bony erosion of the frontal calvarium extending into the bifrontal extra-axial collection. Piperacillin/tazobactam and doxycycline were started for frontal osteomyelitis. The oral and maxillofacial surgery team performed an incision and drainage of the abscess (Figure 7). Wound and blood cultures, as well as, acid-fast bacilli stain, did not exhibit any microbial growth. The patient was discharged home with a six-week course of amoxicillin/clavulanic acid to cover for both gram-positive, negative, and anaerobes. Upon one-month follow-up at the infectious disease clinic, the swelling and erythema had resolved.

## Discussion

Sir Percivall Pott initially reported PPT in connection with sinusitis and head trauma in 1768 [1]. PPT is an enlargement of the forehead, which most commonly occurs secondary to osteomyelitis of the frontal bone, leading to a subperiosteal abscess. Although there are numerous ways that an infection might spread, it often does so either directly across the frontal bone or as thrombophlebitis through the diploic veins. The frontal bone can be destroyed, which can cause significant protrusion of the forehead [2,3]. The subperiosteal abscess forms as a result of purulent erosion through the frontal bone. A further inward extension might cause cerebral problems, including epidural or subdural empyema, meningitis, or abscess [4]. Streptococcal or staphylococcal species, in combination with anaerobes infection, are the most frequent cause of this condition. Most reported cases grew both aerobes and anaerobes organisms. Infections are often polymicrobial in nature [5]. As a result, empiric therapy is advised as the first line of treatment for gram positives and anaerobes, due to the fact that cultures may not grow bacterial organisms if the patient was given antibiotics prior to obtaining cultures.

Risk factors for developing PPT include chronic sinusitis, penetrating deformities (caused by trauma or surgical procedures), dental problems, and cocaine usage. Cocaine and tobacco-induced PPT increases the risk for recurrence as it makes the sinus bones thin and fragile [6].

The typical presentation of PPT is a swollen forehead accompanied by a headache and fever. Individuals frequently have an overall health condition and lack a clear history of prior sinusitis. In immunocompromised patients, uncommon but severe complications like osteomyelitis, orbital cellulitis, meningitis, subdural empyema, brain abscess, and cavernous sinus thrombosis have been mentioned [2,3].

CT and MRI scans are important in diagnosing PPT as they can define the extent of the infection as well as the possible involvement of bone or sinuses [7]. While the CT scan provides in-depth information regarding the integrity of the anterior and posterior bones of the frontal sinus [8], the MRI scan can communicate the state of soft tissue both intracranially and extracranially.

Surgical debridement of the afflicted sinus is the primary definitive treatment for PPT, followed by a lengthy course of antibiotics that should be guided by the growing cultures [9]. Prolonged antibiotics, sinus irrigations, and adequate frontal sinus outflow tract drainage, to prevent mucous or pus accumulation, are important components in treating this entity.

## Conclusions

Although it has been named a tumor, PPT is a subperiosteal abscess secondary to osteomyelitis of the frontal bone. It is more frequent in children and rarely occurs in adults. Causes include but are not limited to head trauma, chronic sinusitis, the most likely cause in our case, or cocaine use. Accurate diagnosis and aggressive management can prevent complications like epidural empyema, infraorbital abscess, and cavernous sinus thrombosis. Hematogenous spread to dural venous sinuses, which drain the frontal sinus through diploic veins, can cause sepsis and death. CT scan can establish a diagnosis and identifies the progression of the infection. Adolescents are the most commonly affected patient population; however, it can affect any age, from preadolescents to the elderly, as in our case. With the availability of antibiotics, the prognosis of PPT has decreased significantly. After appropriate antibiotics and sinus drainage, complete resolution of sinusitis and radiological follow-up is crucial due to the possible recurrence of the pathology. Our case sheds light on the rarity of PPT, the possible involvement of any age, and the current management of this entity.
